# Supplementary figures and images for: Rhizobacterial communities of five co-occurring desert halophytes
Source: PeerJ. 2018 Aug 30;6:e5508. doi: 10.7717/peerj.5508 (PMC6119601; doi:10.7717/peerj.5508)

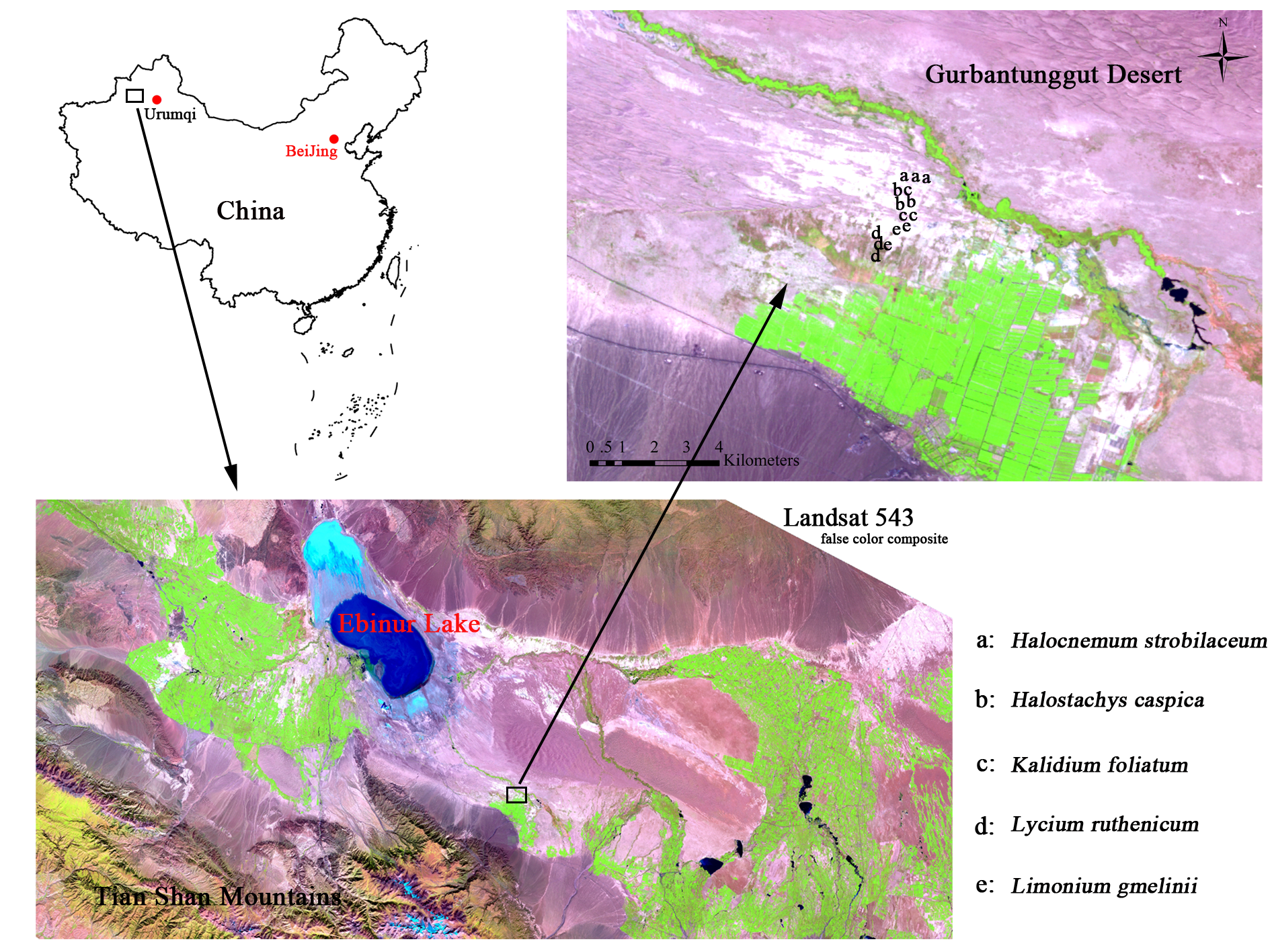

Supplement: Figure S1 — The maps were downloaded from Google Maps. [file peerj-06-5508-s001.png]

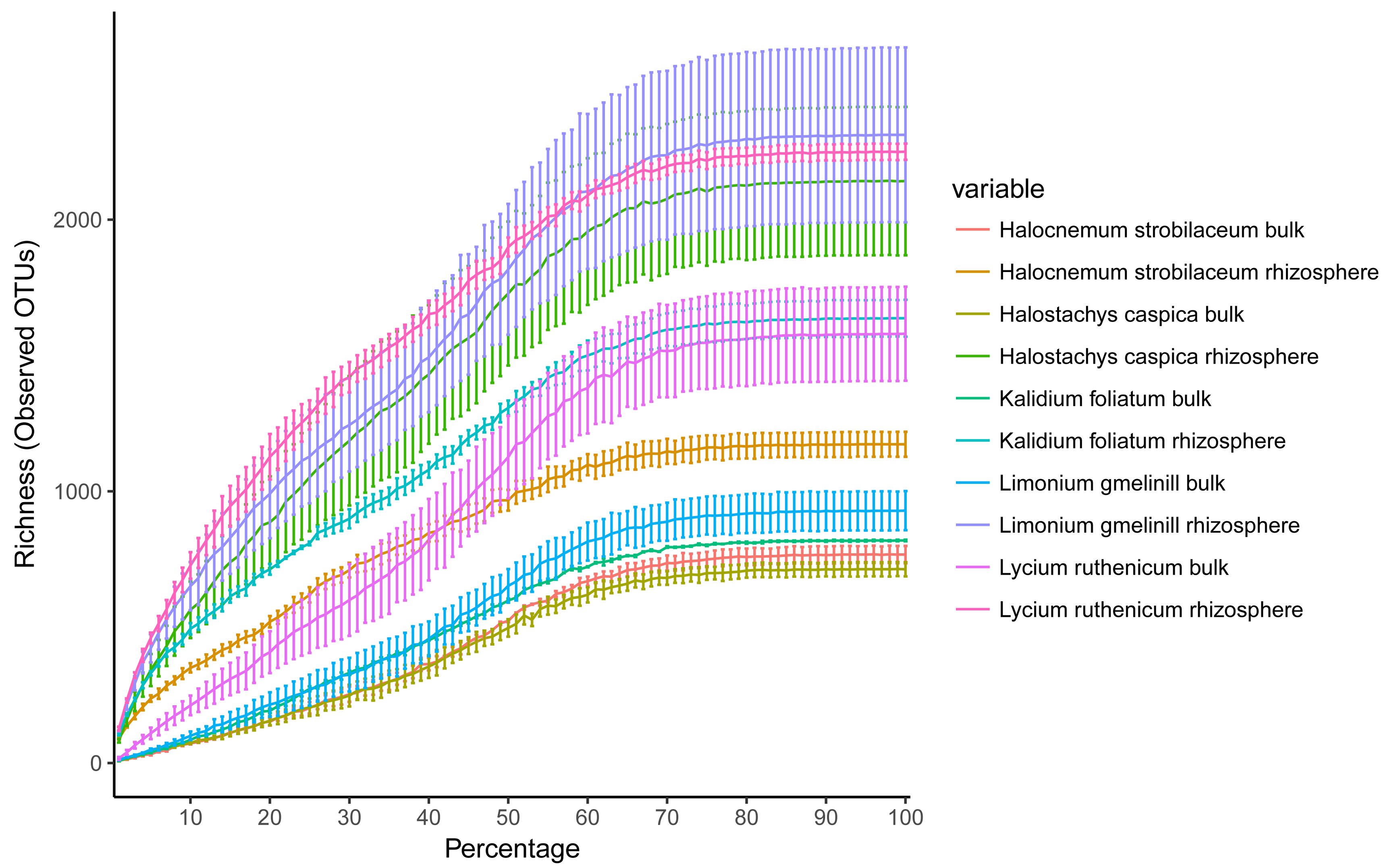

Supplement: Figure S2 — Error bars indicate standard errors. [file peerj-06-5508-s002.png]

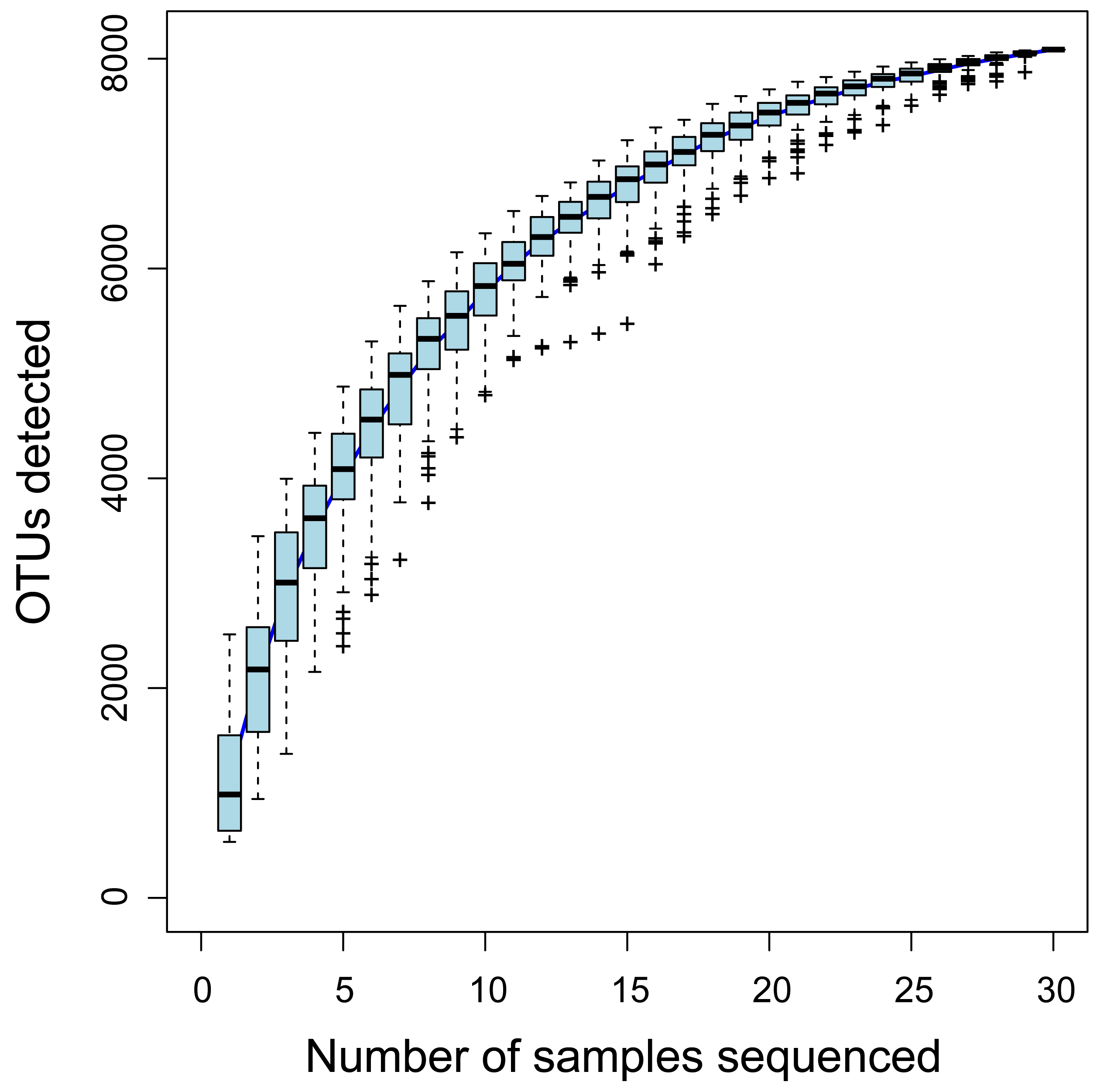

Supplement: Figure S3 — The horizontal bars within boxes represent the medians, while the tops and bottoms of the boxes represent 75th and 25th quartiles, respectively. Plus signs(+) indicate outliers. [file peerj-06-5508-s003.png]

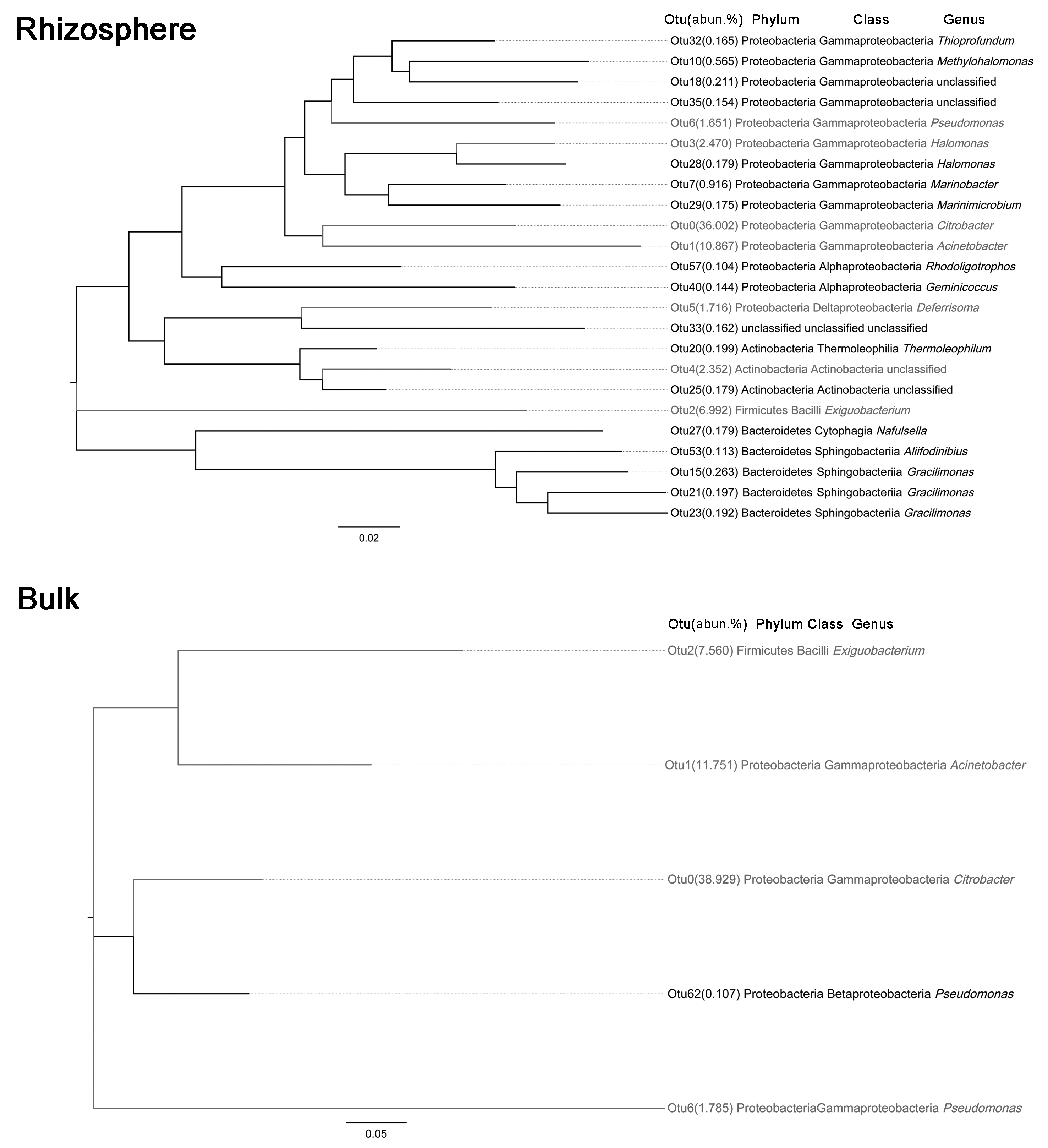

Supplement: Figure S4 [file peerj-06-5508-s004.png]

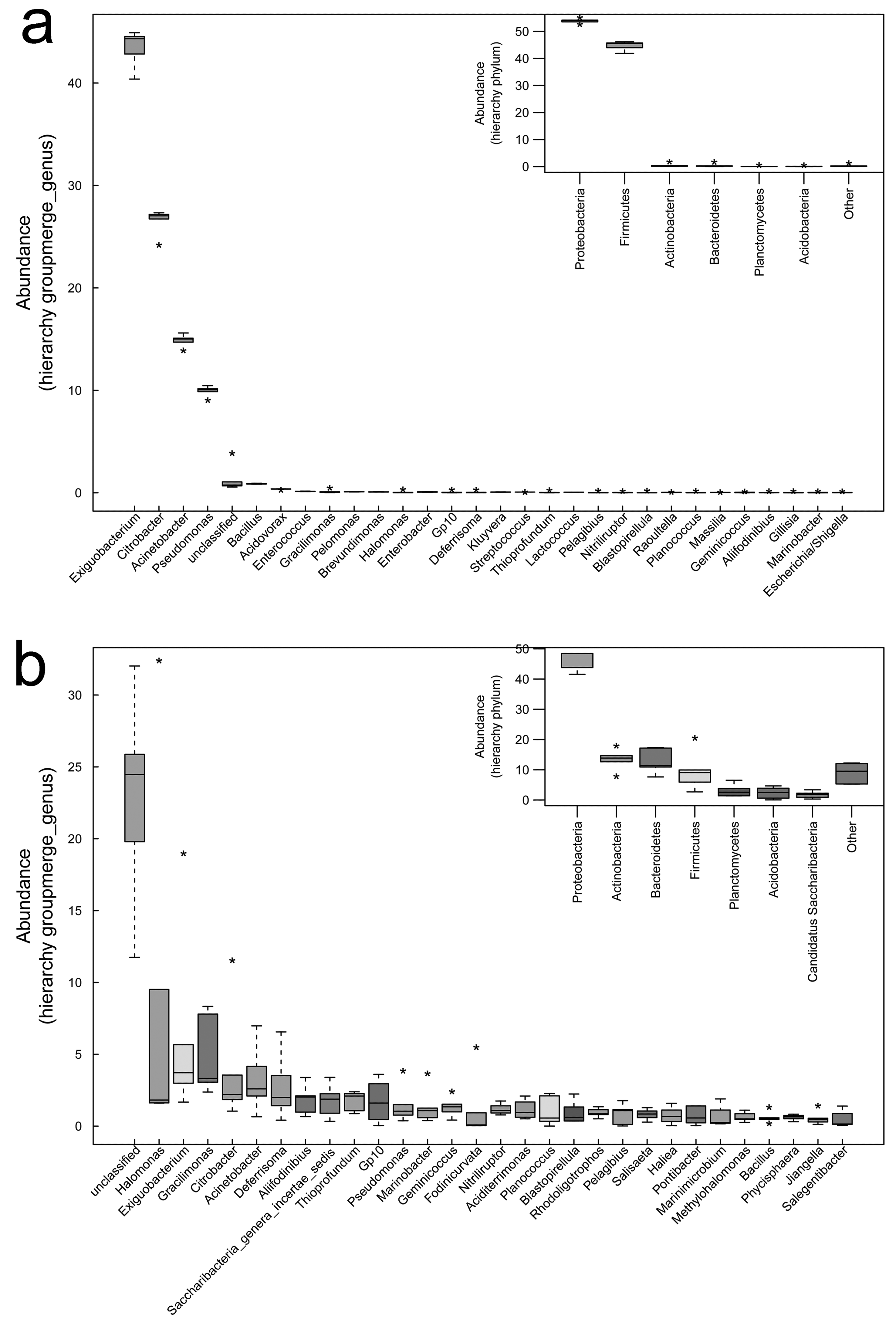

Supplement: Figure S5 [file peerj-06-5508-s005.png]
