# Supplementary material for: Rhizobacterial communities of five co-occurring desert halophytes
Source: PeerJ. 2018 Aug 30;6:e5508. doi: 10.7717/peerj.5508 (PMC6119601; doi:10.7717/peerj.5508)
Supplement: Table S1 [file peerj-06-5508-s006.docx]

**Table S1** Alpha diversity indices of bacterial communities in the rhizosphere and bulk soils.

|  | Rhizosphere | | | | | Bulk | | | | |
| --- | --- | --- | --- | --- | --- | --- | --- | --- | --- | --- |
| Species | OTUs  (total in all replicates/shared by all replicates) | OTUs richness | Shannon index | ACE index | Good's coverage | OTUs  (total in all replicates/shared by all replicates) | OTUs richness | Shannon index | ACE index | Good's coverage |
| *Lycium ruthenicum* | 4272/729 | 2249 ± 52 | 5.83 ± 0.23a | 3108.01 ± 101 | 0.98 ± 0.00 | 3688/220 | 1579 ± 299 | 2.52 ± 0.39a | 4085.66 ± 871 | 0.98 ± 0.00 |
| *Limonium gmelinii* | 4602/665 | 2312 ± 557 | 5.60 ± 0.51a | 4053.22 ± 1138 | 0.98 ± 0.00 | 2152/152 | 929 ± 124 | 2.0 ± 0.1b | 2626.43 ± 1116 | 0.99 ± 0.00 |
| *Kalidium foliatum* | 3336/442 | 1637 ± 116 | 5.11 ± 0.11b | 2811.08 ± 725 | 0.99 ± 0.00 | 1801/107 | 819 ± 5 | 1.83 ± 0.03b | 1685.14 ± 129 | 0.99 ± 0.00 |
| *Halostachys caspica* | 4291/517 | 2142 ± 472 | 4.73 ± 0.80b | 3523.5 ± 156 | 0.98 ± 0.01 | 1699/94 | 713 ± 46 | 1.76 ± 0.05b | 2090.46 ± 156 | 0.99 ± 0.00 |
| *Halocnemum strobilaceum* | 2342/317 | 1173 ± 79 | 4.19 ± 0.30b | 1684.03 ± 949 | 0.99 ± 0.00 | 1108/112 | 767 ± 55 | 1.77 ± 0.04b | 2378.62 ± 1195 | 0.99 ± 0.00 |
| mean | - | 1903 ± 531 | 5.07 ± 0.66 | 3035.97±888 | 0.98 ± 0.00 | - | 961 ± 351 | 1.95 ± 0.31 | 2573.26±915 | 0.99 ± 0.00 |

Values are given as means ± SD (n = 3).

Different letters indicate significant differences among five halophytes (*P* < 0.05).
